# Enhancing the Oxidation of Toluene with External Electric Fields: a Reactive Molecular Dynamics Study

**DOI:** 10.1038/s41598-017-01945-4

**Published:** 2017-05-10

**Authors:** Shen Tan, Tao Xia, Yao Shi, Jim Pfaendtner, Shuangliang Zhao, Yi He

**Affiliations:** 1 College of Chemical and Biological Engineering, Zhejiang University, Hangzhou, 310027 P. R. China; 2 Key Laboratory of Biomass Chemical Engineering of Ministry of Education, Hangzhou, P. R. China; 3 Department of Chemical Engineering, University of Washington, Seattle, Washington 98105 United States; 4 School of Chemical Engineering, East China University of Science and Technology, Shanghai, 200237 P. R. China

## Abstract

The effects of external electric field (Efield) on chemical reactions were studied with the reactive molecular dynamics (ReaxFF MD) simulations by using the oxidation of toluene as a model system. We observed that Efields may greatly enhance the oxidation rate of toluene. The initial reaction time of toluene is also reduced remarkably in Efields. A stronger Efield leads to a faster oxidation rate of toluene. Further studies reveal that the applying of a Efield may result in the oxidation of toluene at 2100 K which is otherwise not able to happen when the Efield is not present. The oxidation rate of toluene at 2100 K in a Efield is comparable with the oxidation rate of toluene at 2900 K when the Efield is not applied. In addition, Efields were observed to significantly enhance the occurrence of the initial radical generation for different pathways of toluene oxidation but they do not seem to favor any of the pathways. Finally, Efields do not seem to enhance the polarization of toluene during its transition state, which suggests that a polarizable charge equilibration method (PQEq) method might be needed to take the effects of Efields into consideration.

## Introduction

Tuning chemical reactions with new and more-efficient ways has long been a desirable goal for researchers^1, 2^. Inoue^3^ measured the oxidation rate of toluene in an electrical discharge as early as 1955. In 1991, Eriksson^4^ further examined the same reaction and observed that a 50 Hz electrical discharge can affect toluene oxidation. Chemical reactions in general can be viewed as the movement of electrons and/or nuclei. Therefore, one might expect that their kinetics could be influenced by external electric fields (Efields). Theoretical studies^5–7^ with Quantum Mechanics (QM) predicted that Efields could in principle affect the stability of chemical species by stabilizing or destabilizing charge-separated resonance contributors to control the total reaction process which is often referred to as electrostatic catalysis. In consistent with the theoretical studies above, Boxer^8^ reported that atoms in the active site of enzymes could impose specific electrostatic fields onto their bound substrates. The magnitude of the field exerted by the active site strongly correlates with the catalytic rate enhancement of enzyme. In another study, Warshel^9^ proposed that electrostatic transition-state stabilization is the most important factor in enzyme catalysis and demonstrated that enzyme active sites provide a preorganized polar environment that stabilizes the transition state much more than the corresponding environment in water. Other theoretical QM calculations^10–12^ and experimental studies^13–15^ also showed that the reactivity or selectivity of chemical reactions could be controlled by Efields. For instance, Aragonès^2^ presented the first experimental evidence of a non-redox, bond-forming process of Diels–Alder reaction being accelerated by an oriented Efield. The experimental results are qualitatively consistent with their quantum chemistry calculations and the similar calculations reported by Meir^10^, validating that the ability of the Efield affects stabilities of chemical species via a minor chargeseparated resonance contributor of the transition state.

Most currently theoretical studies on the effect of Efields on chemical reactions use QM as the primary tool. While QM provide lots of critical information about chemical reactions such as the barrier height for certain chemical reaction^10^, the stabilities of chemical species^6^, participation in bond-forming and bond-breaking steps^7^ and so on. Unfortunately, the computational cost inherent to QM level calculations severely limits the scales of these simulations. This limitation often excludes QM approaches from being considered for the studies of the dynamic evolution of chemical reactions, thus hampering the theoretical understanding of key factors affecting the overall reaction system. On the other hand, classic molecular dynamics (MD) simulations are suitable for the studies on a system in large scales, however, they are usually incapable of describing the evolution of a system involving chemical reactions.

The reactive force field (ReaxFF)^16, 17^ methodology, developed by van Duin, Goddard, and co- workers, has opened the new door for numerous studies of phenomena occurring on scales that were previously inaccessible to computational methods. In this approach, QM structure and energy data are used to train the empirical ReaxFF that require significantly fewer computational resources, thereby enabling simulations to better describe dynamic processes. ReaxFF is based on a general bond order/bond distance relationship introduced by Tersoff^18^, and the van der Waals and Coulomb interactions are explicitly taken into account. In ReaxFF, the general energy function takes the following calculation by equation (1)^17^.

1 $${{\rm{E}}}_{{\rm{system}}}={{\rm{E}}}_{{\rm{bond}}}+{{\rm{E}}}_{{\rm{over}}}+{{\rm{E}}}_{{\rm{under}}}+{{\rm{E}}}_{{\rm{val}}}+{{\rm{E}}}_{{\rm{pen}}}+{{\rm{E}}}_{{\rm{tors}}}+{{\rm{E}}}_{{\rm{conj}}}+{{\rm{E}}}_{{\rm{vdWaals}}}+{{\rm{E}}}_{{\rm{coulomb}}}$$

where E_bond_ denotes the bond energy, E_over_ and E_under_ represent the over-coordinated atom and under-coordinated atom in the energy contribution, respectively. Other terms, including E_val_, E_pen_, E_tors_, E_conj_, E_vdWaals_ and E_Coulomb_ are the valence angle term energy, penalty energy, torsion energy, conjugation effects to molecular energy, nonbonded van der Waals interaction and Coulomb interaction, respectively.

The ReaxFF was first developed for hydrocarbons. Since then, it was also used to analyze the hydrocarbon oxidation^19–21^, material properties^22, 23^, and other complicated chemical reactions such as the pyrolysis and oxidation of benzene^24^, toluene^25^ or n-dodecane^26^. For instance, Chenoweth^19^ and coworkers investigated the initiation mechanisms and kinetics associated with the pyrolysis of JP-10 (exotricyclo[5.2.1.0^2, 6^]decane), a single-component hydrocarbon jet fuel with ReaxFF simulations. ReaxFF MD provides good agreement with experiments for the product distribution as a function of temperature. Cheng *et al*.^25^ performed the ReaxFF reactive force field to study the high-temperature oxidation mechanisms of toluene at different temperatures and densities. Meanwhile, they further studied the details of kinetic analysis of the oxidation of toluene and obtained good Arrhenius behaviors. Besides, the fitted apparent activation energies agree well with the experimental results, suggesting that ReaxFF can provide accurate analysis in terms of the oxidation processes of toluene. The main difference between traditional unreactive force fields and ReaxFF is that in ReaxFF, the connectivity is determined by bond orders calculated from interatomic distances that are updated in every MD step. This allows for bonds to break and form during the simulations. In the meantime, the computing cost of ReaxFF is comparable with the classical molecular dynamics. Furthermore, it can achieve an accuracy comparable to quantum mechanical methods when analyzing some large scale chemical reactions so it is reliable to study chemical reaction process and products.

In this work, we used the oxidation of toluene as a model system to examine the effects of Efields^27–29^ on chemical reactions. Strong Efields were applied to the system so that the oxidation can be observed in a relatively short simulation time. The oxidation of toluene with/without Efield were compared. Detailed studies on effects of Efields at different strength, different densities and temperatures of systems were carried out. In addition, the oxidation mechanisms of toluene and the partial charge of atoms in toluene at its transition state were also investigated.

## Results

### The toluene oxidation with/without Efields

**Figure 1:**
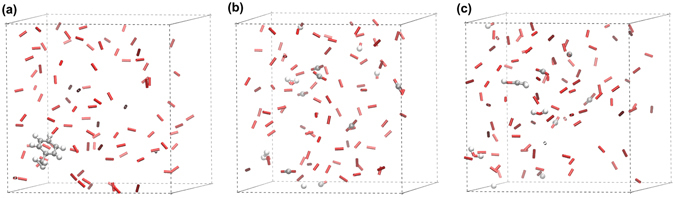
(**a**) Initial configuration of the equilibrated toluene/oxygen system (T = 2500 K, density = 0.15 g/cm^3^). (**b**) Final configuration after a 3000 ps NVT-MD simulation (T = 2500 K, density = 0.15 g/cm^3^, Efield = 0.2 V/Å) was performed. (**c**) Final configuration after a 3000 ps NVT-MD simulation (T = 2500 K, density = 0.15 g/cm^3^, Efield = 0 V/Å) was performed. Molecular oxygen is represented by sticks and toluene and oxidation products are represented using the ball and stick model.

After examining ten systems with Efield applied and another ten systems without the existence of Efield at the same temperature of 2500 K and density of 0.15 g/cm^3^, we observed that the toluene molecule was oxidized in all the systems with Efields. The toluene molecule was completely transformed into CO_2_, CO, and H_2_O with Efield within 3000 ps as shown in Fig. 1(b). As a comparison, the toluene molecule was oxidized in only three out of ten systems, which is somehow different to similar experimental work of Erkisson and coworkers’^4^, where, instead of using static Efileds, they employed a 50 Hz electric discharge to facilitate toluene oxidation. They have shown that the thermal reaction is negligible in the absence of an electrical discharge. Moreover, there are some intermediates existing even after a 3000 ps simulation for the system where toluene was reacted (Fig. 1(c)). This suggests that Efields may have a significant influence on the oxidation of toluene.

**Figure 2:**
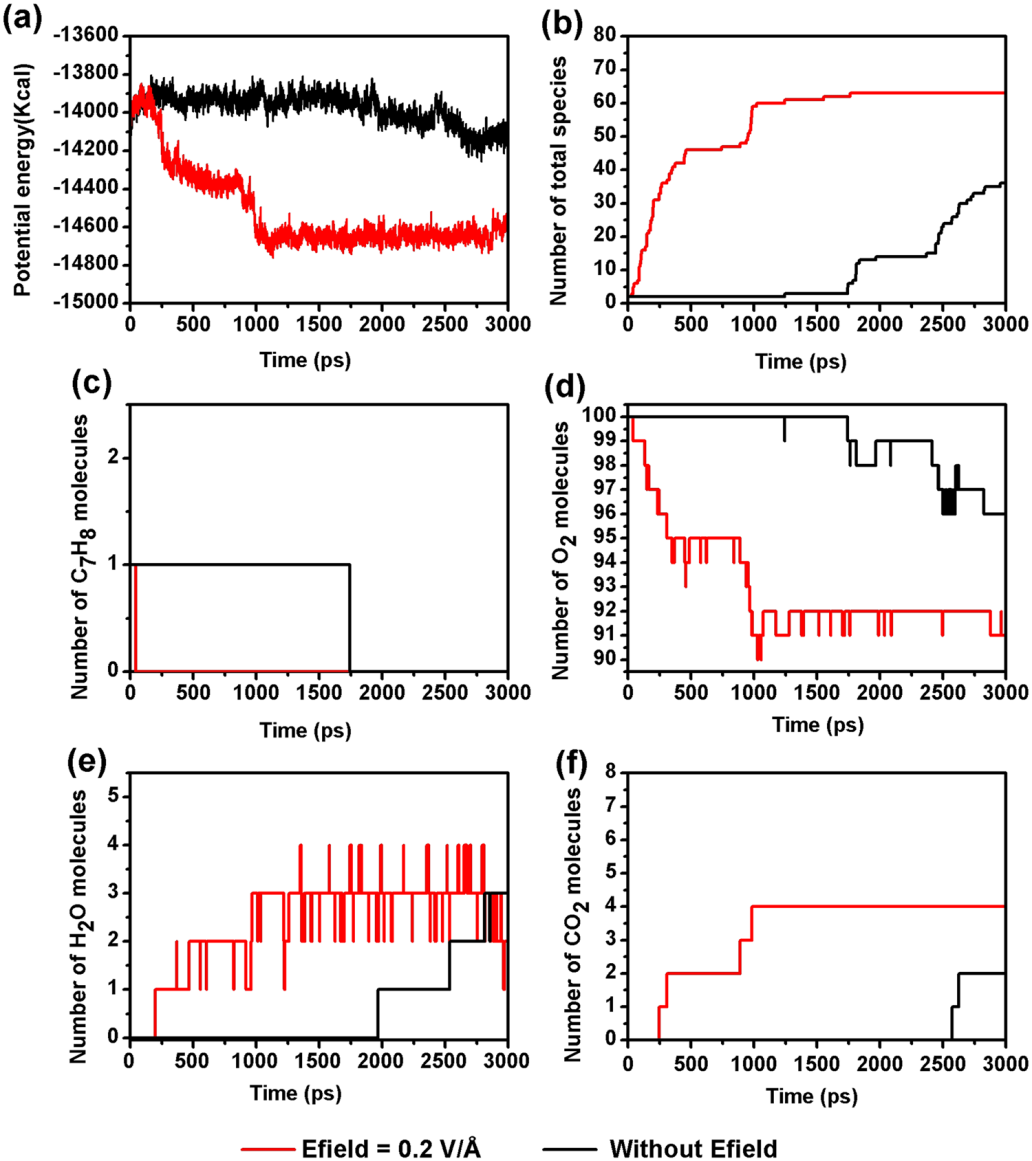
Evolution of the potential energies, total number of species, toluene, oxygen and main products at Efields = 0, 0.2 V/Å (ReaxFF NVT-MD simulation, T = 2500 K, ρ = 0.15 g/cm^3^, toluene/oxygen = 1:100).

Figure 2 exhibits the evolution of representative systems for the oxidation of toluene with/without Efield after examining the trajectory of systems which were saved in every 1 ps. The potential energy of the system, the number of total species, reactants, and products were plotted as a function of time. Systems in the absence/presence of Efields both displayed a similar trend as time passes. However, the system with Efields applied shows significant kinetic enhancements for all these characteristics examined. Figure 2a describes the potential energy evolution of the two systems with/without Efields. As both systems start with the same configuration, the initial potential energies are same to each other. It is known experimentally that the oxidation process of toluene starts with endothermic structural changes before decomposition and followed by exothermic reactions^30^. Therefore, for both of the systems, we observed that an increase in the potential energy at the beginning which is then followed by a decrease. However, the system with Efield exhibits a short period of increase in potential energy which was immediately followed by a sharp decrease. As a comparison, the potential energy of the system without Efield stays at a higher level than its initial one before the toluene finally decomposes at 1700 ps around. Figure 2(b–f) suggests that the first step of the oxidation of toluene is one of the rate limiting steps. Once the toluene starts to be reacted, a cascade of chemical reaction will follow immediately. In addition, Efield seems to greatly enhance the appearance of hydroxyl (OH) and hydroperoxide (HO_2_) radicals, leading to the spikes in Fig. 2(e). These OH and HO_2_ radicals are often observed in water vapor involved chemical reactions^20^.

### The oxidation of toluene in Efields at various toluene densities

**Figure 3:**
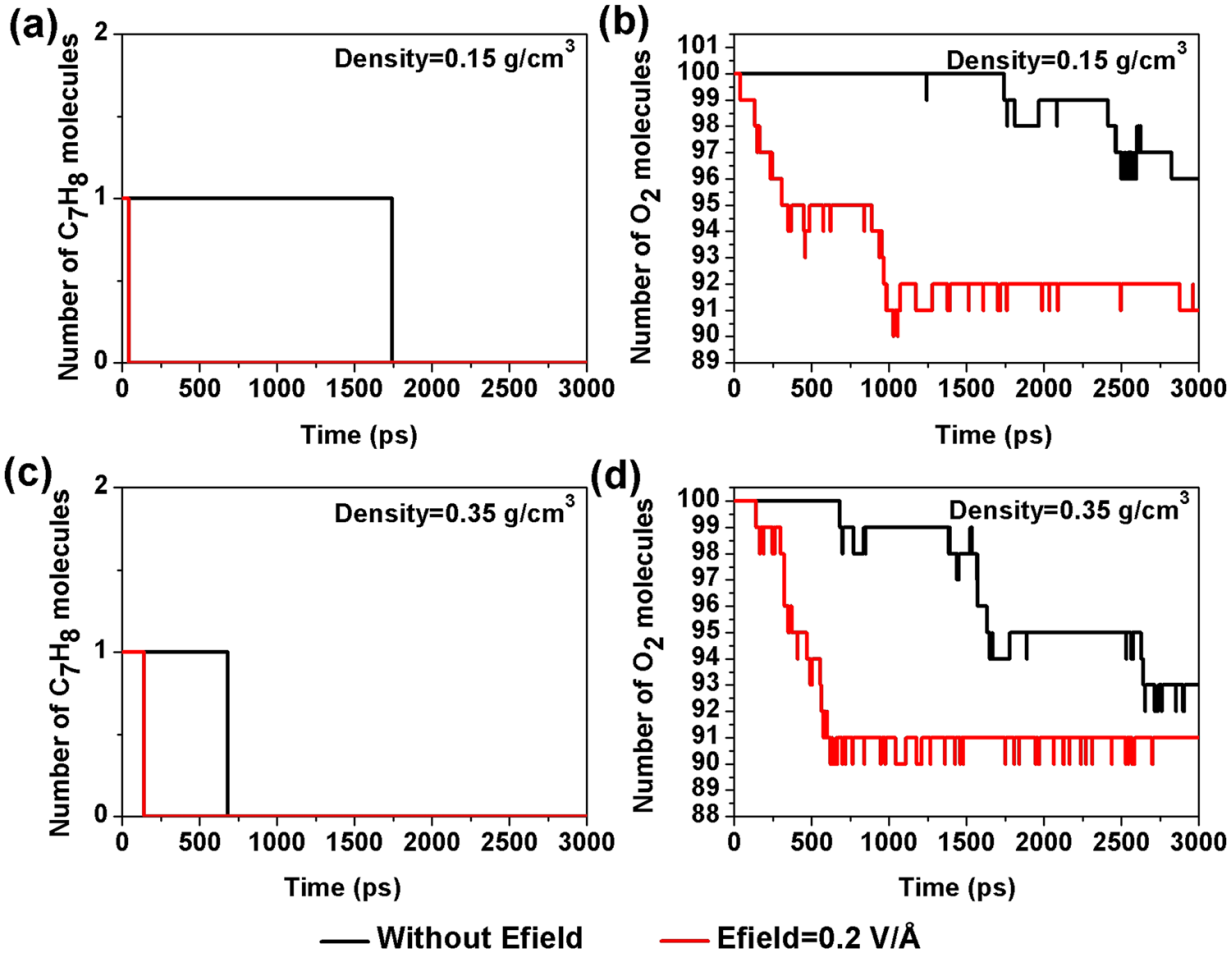
Evolution of the toluene and oxygen molecules at density ρ = 0.15, 0.35 g/cm^3^, Efield = 0, 0.2 V/Å (ReaxFF NVT-MD simulation, T = 2500 K, toluene/oxygen = 1:100).

The effect of toluene density on its oxidation rate was investigated by changing the size of the simulation box. There are two densities examined in this work, which corresponds to a density of ρ = 0.15 g/cm^3^ and 0.35 g/cm^3^ for the modeling systems. We also studied ten different initial configurations with/without a Efield of 0.2 V/Å applied. Each configuration still contains one toluene molecules and one hundred oxygen molecules. Figure 3 displays that there is significant difference in terms of the oxidation rate of toluene when the density of toluene is increased from 0.15 g/cm^3^ to 0.35 g/cm^3^ when no Efield is applied. Higher toluene density gives a significant boost to its oxidation rate, which is in agreement with previous studies^25^. However, when applying a Efield of 0.2 V/Å, there is no much difference in the oxidation rate of toluene, as the oxidation rate of toluene has already been greatly enhanced.

**Figure 4:**
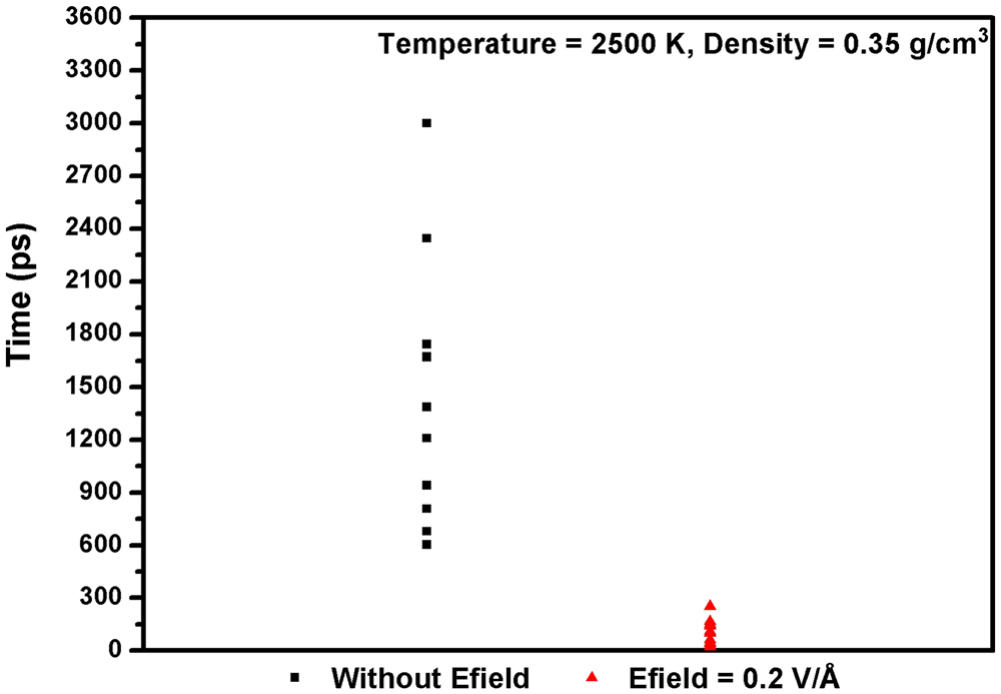
Initial reaction time with/without Efield at T = 2500 K, density ρ = 0.35 g/cm^3^ extracted from ReaxFF NVT-MD simulations.

In addition, we also examined the reactivity of toluene by calculating its initial reaction time. The initial reaction time is denoted as the time required for observation of the first reaction in the simulation that coincides with the disappearance of the original toluene molecule. Because the toluene molecule was oxidized in only three out of ten systems without Efields at density of 0.15 g/cm^3^ in 3000 ps simulation time we observed, we only calculated the initial reaction time in simulations at density of 0.35 g/cm^3^. The results in Fig. 4 show that for systems with Efields, the initial reaction time of toluene collapses into a small range of time at 2500 K, which varies from 20–250 ps. For the systems without any applied Efield, there is a broad distribution for the initial reaction time of toluene, ranging from 600 ps to over 3000 ps, which they most likely located in between 600–900 ps. As both oxidation process of toluene and o-xylene^20^ has similar mechanism and bond dissociation energy^31^ for its first step, the initial reaction time of toluene is comparable to that of o-xylene, which is 748 ps reported by van Duin and his coworkers^20^. It seems that when a Efield is applied, the initial reaction time for oxidation of toluene can be significantly reduced.

### The oxidation of toluene in Efields with various strength

**Figure 5:**
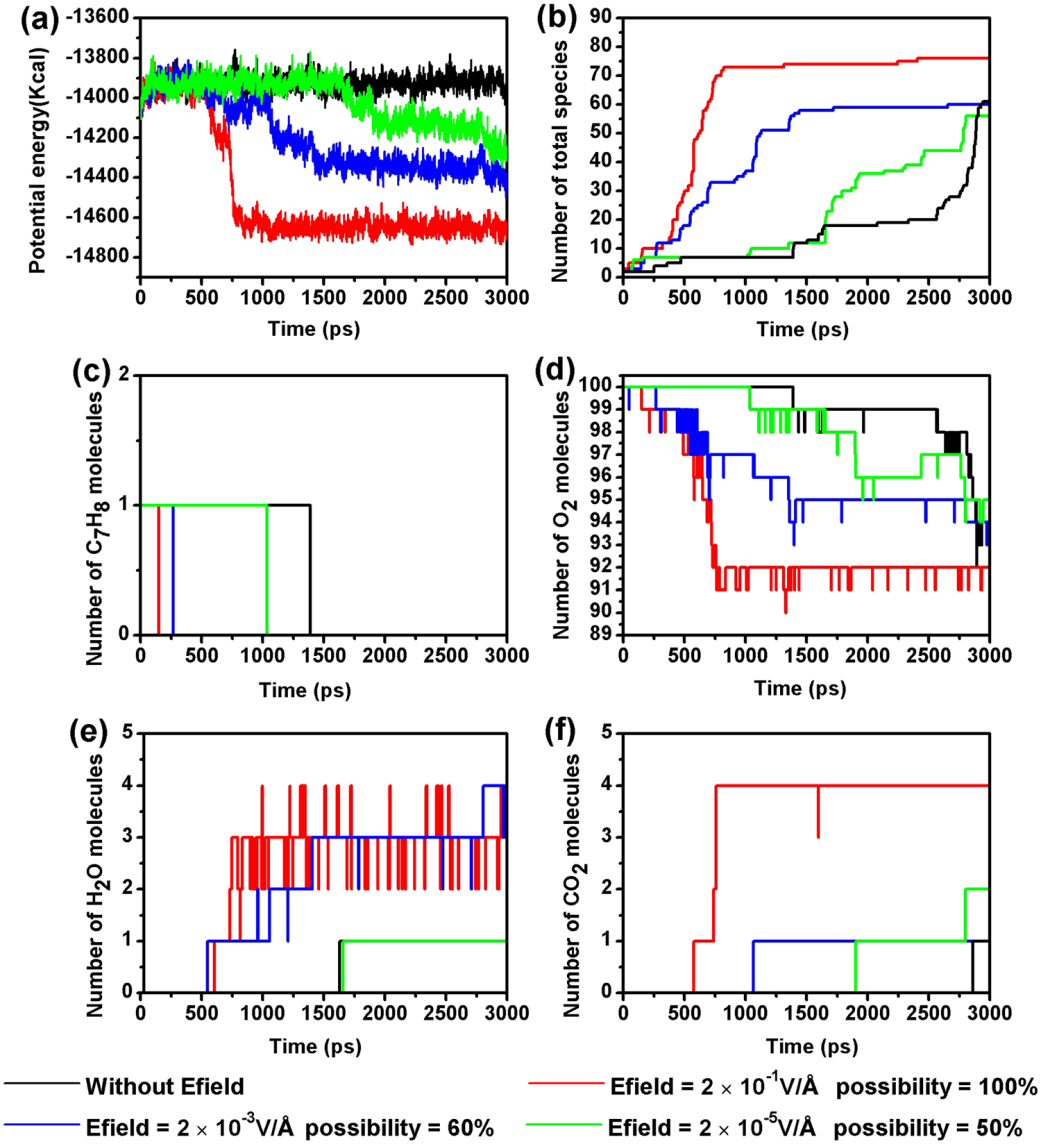
Evolution of the potential energies, total number of species, toluene, oxygen and main products at Efield = 2 × 10^−1^, 2 × 10^−3^, 2 × 10^−5^ and 0 V/Å (ReaxFF NVT-MD simulation, ρ = 0.35 g/cm^3^, T = 2500 K, toluene/oxygen = 1:100).

The effects of Efield were further analyzed by performing a series of NVT-MD simulations with a Efield strength of 2 × 10^−5^, 2 × 10^−3^, 2 × 10^−1^ and 0 V/Å, respectively. For each Efield strength, we ran ten simulations with different initial configurations. The representative curves for the evolution of potential energy, total number of species, reactants, and products are displayed in Fig. 5. The curves demonstrate that Efield with high strength leads to faster an oxidation of toluene. In addition, we observed that at 100% of the ten simulations ended up with curves similar to the representative curves in Fig. 5(a–f) when a Efield strength of 2 × 10^−1^ V/Å was applied. When the strength of the Efield decreases, the possibility of observing the representative curves in Fig. 5(a–f) is significantly reduced. For instance, only 50% of the simulation have curves similar to the representative curves in Fig. 5(a–f) for the Efield of 2 × 10^−5^ V/Å.

### Comparison between the effects of Efields and temperature on toluene oxidation

**Figure 6:**
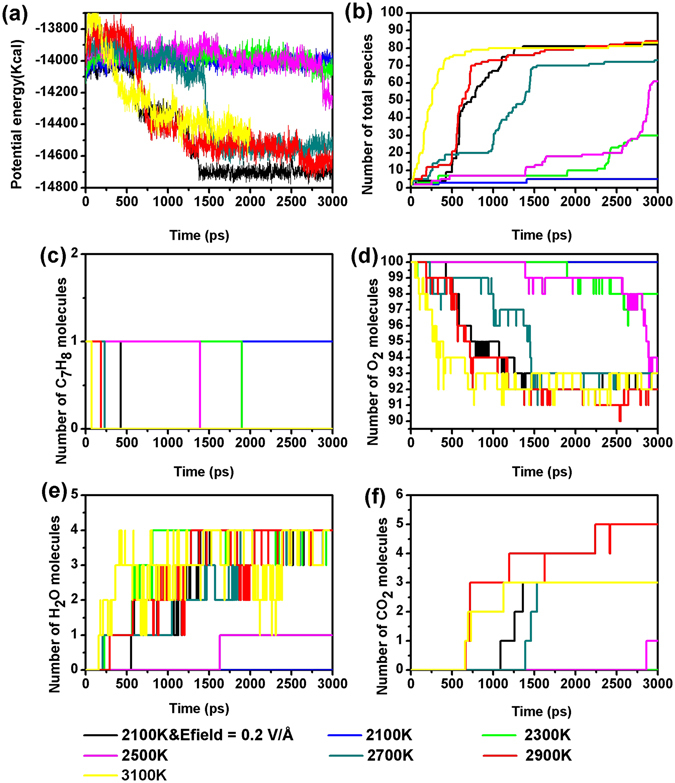
Evolution of the potential energies, total number of species, toluene, oxygen and main products at T = 2100 K, 2300 K, 2500 K, 2700 K, 2900 K, 3100 K as well as T = 2100 K and Efield = 0.2 V/Å (ReaxFF NVT-MD simulation, ρ = 0.35 g/cm^3^, toluene/oxygen = 1:100).

To compare the effects of Efield and temperature on toluene oxidation, a series of NVT–MD simulations with/without Efield are performed at temperatures from 2100 to 3100 K at an interval of 200 K. Figure 6 shows the evolution of the potential energy of the system, total number of species, reactants, and products at a density of 0.35 g/cm^3^. It can be obviously seen that the rate of toluene oxidation reaction increases as the temperature gets higher, which is in a good agreement with previous studies^25, 26^. When the temperature is set to 2100 K, no oxidation of toluene was observed during the period of the simulations due to the poor reactivity. Interestingly, when a Efield is applied to such system at 2100 K, we observed a fast oxidation rate of toluene which is comparable to the system at 2900 K. Therefore, the results show that by applying Efields, the required temperature for a reaction may be significantly reduced, in consistent with previous experimental research by Eriksson and coworkers^4^.

### Reaction pathways of toluene oxidation in Efields

**Figure 7:**
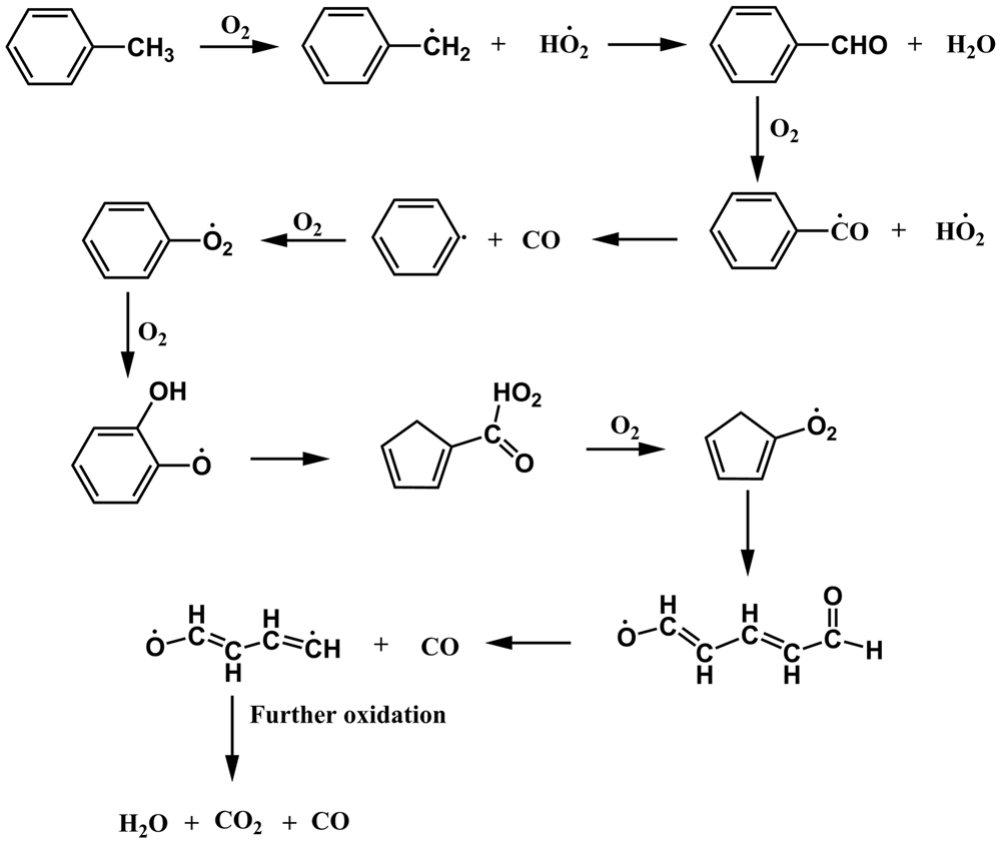
Toluene oxidation pathway through hydrogen abstraction reactions observed during ReaxFF NVT-MD simulations.

**Figure 8:**
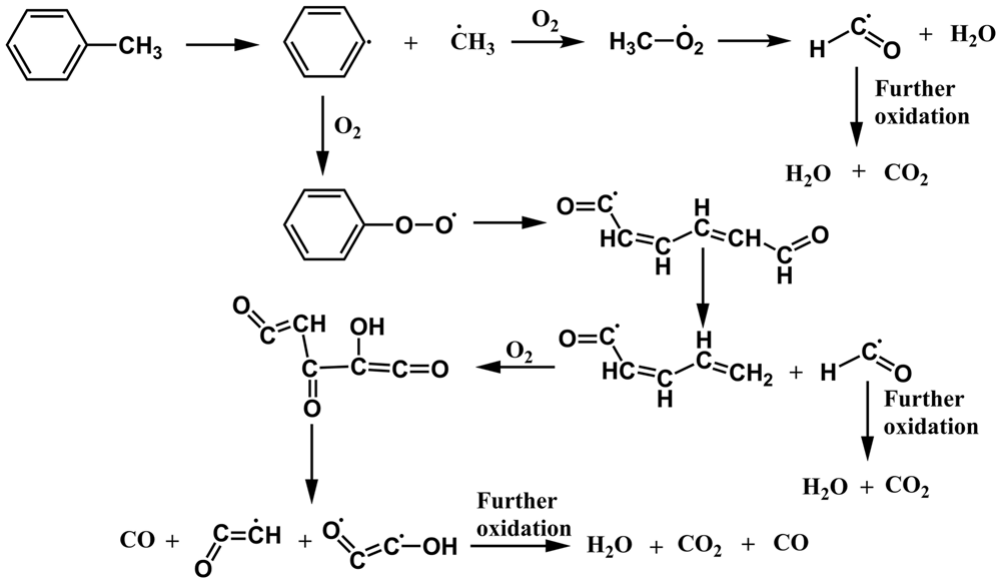
Toluene oxidation pathway through pyrolysis observed during ReaxFF NVT-MD simulations.

To understand how Efield can accelerate the oxidation of toluene, detailed studies on reaction pathways and free radical productions were carried out. The simulations were performed at 2500 K with an Efield of 0.2 V/Å. Ten initial configurations were used for the simulations. It’s known from previous simulation studies^25^ that there are two representative pathways for toluene oxidation: one starts with the abstraction of hydrogen-atom from toluene with the help of oxygen, the other starts with the pyrolysis of toluene. These two pathways are both observed in our simulations. They have been also reported in other experimental^32, 33^ and kinetic modeling studies^34, 35^. Detailed chemical reactions for each pathways are shown in Figs 7 and 8, respectively. Simulation results also show that, among the ten initial configurations we studied, nine of them follows the reaction pathway similar to Fig. 7, while only one follows the reactions in Fig. 8. This is because Fig. 8 has a higher reaction barrier(103.72 kcal/mol) than Fig. 7 does (41.12 kcal/mol) for the first step of the pathways, which is studied by Baulch and his coworkers^36^. This partition of reaction pathway is similar to the oxidation of toluene without the existence of Efields. Therefore, it seems that Efields do not make observable changes in the oxidation mechanism of toluene.

**Figure 9:**
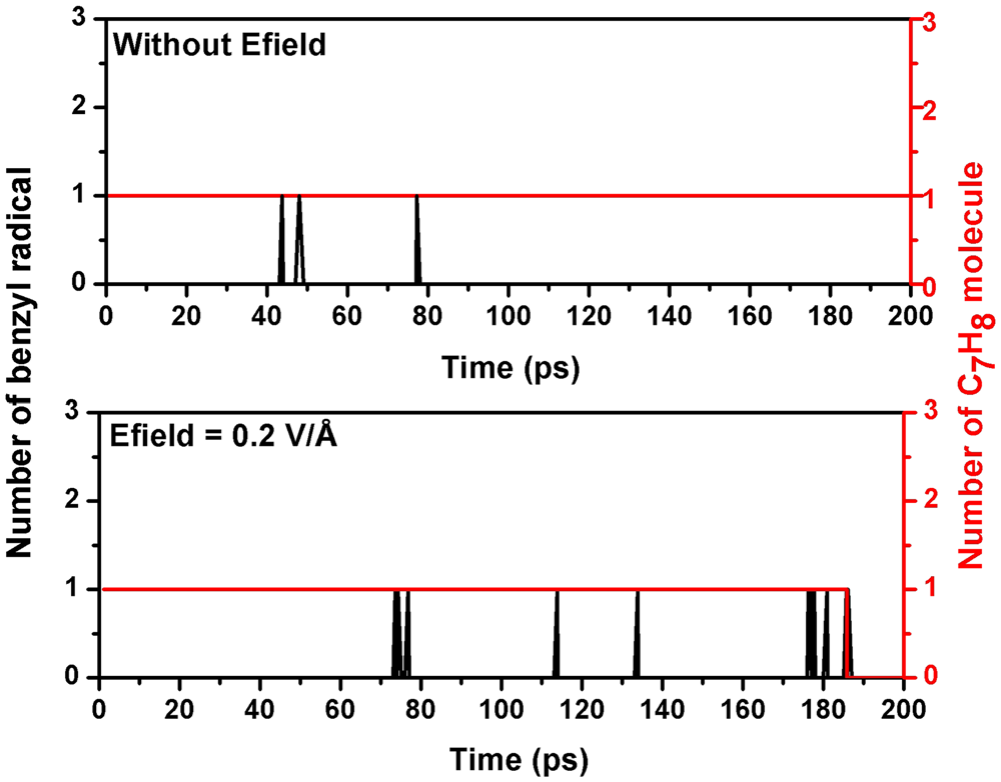
Production of benzyl radicals obtained during ReaxFF NVT-MD simulations with/without Efield = 0.2 V/Å.

**Figure 10:**
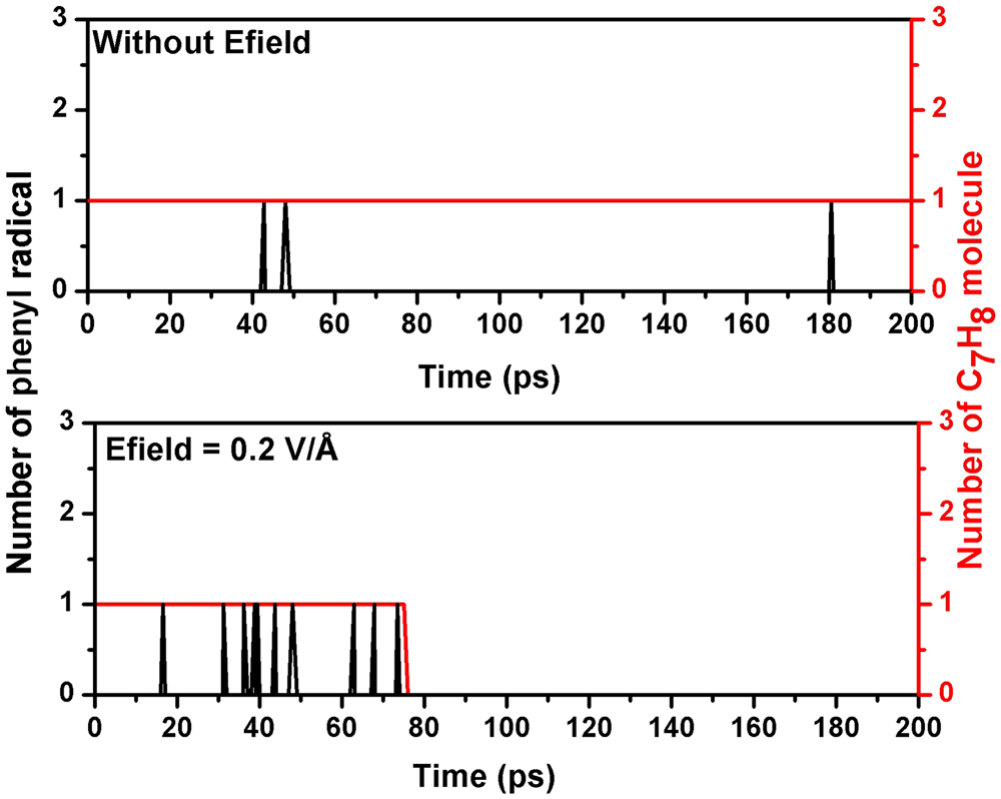
Production of phenyl radicals obtained during ReaxFF NVT-MD simulations with/without Efield = 0.2 V/Ǻ.

For Fig. 7, the hydrogen abstraction in the toluene molecule by oxygen is the initial step of oxidation of toluene, which leads to a new hydroperoxyl radical^20^ and a benzyl^37^. Then we analyzed the production of benzyl radical in simulations with/without Efield after examining the trajectory of systems which were saved in every 50 fs during the initial 200 ps. The spikes in Fig. 9 represents the occurrence of benzyl radical during the oxidation of toluene. The existence of Efields significantly enhance the occurrence of benzyl radical, reduce the initial reaction time, which eventually leads to a faster decomposition of toluene. Similar mechanisms can be used to explain the oxidation rate enhancement of Efield for Fig. 8, as revealed by Fig. 10.

### Partial charge of atoms in Efields at transition states

**Figure 11:**
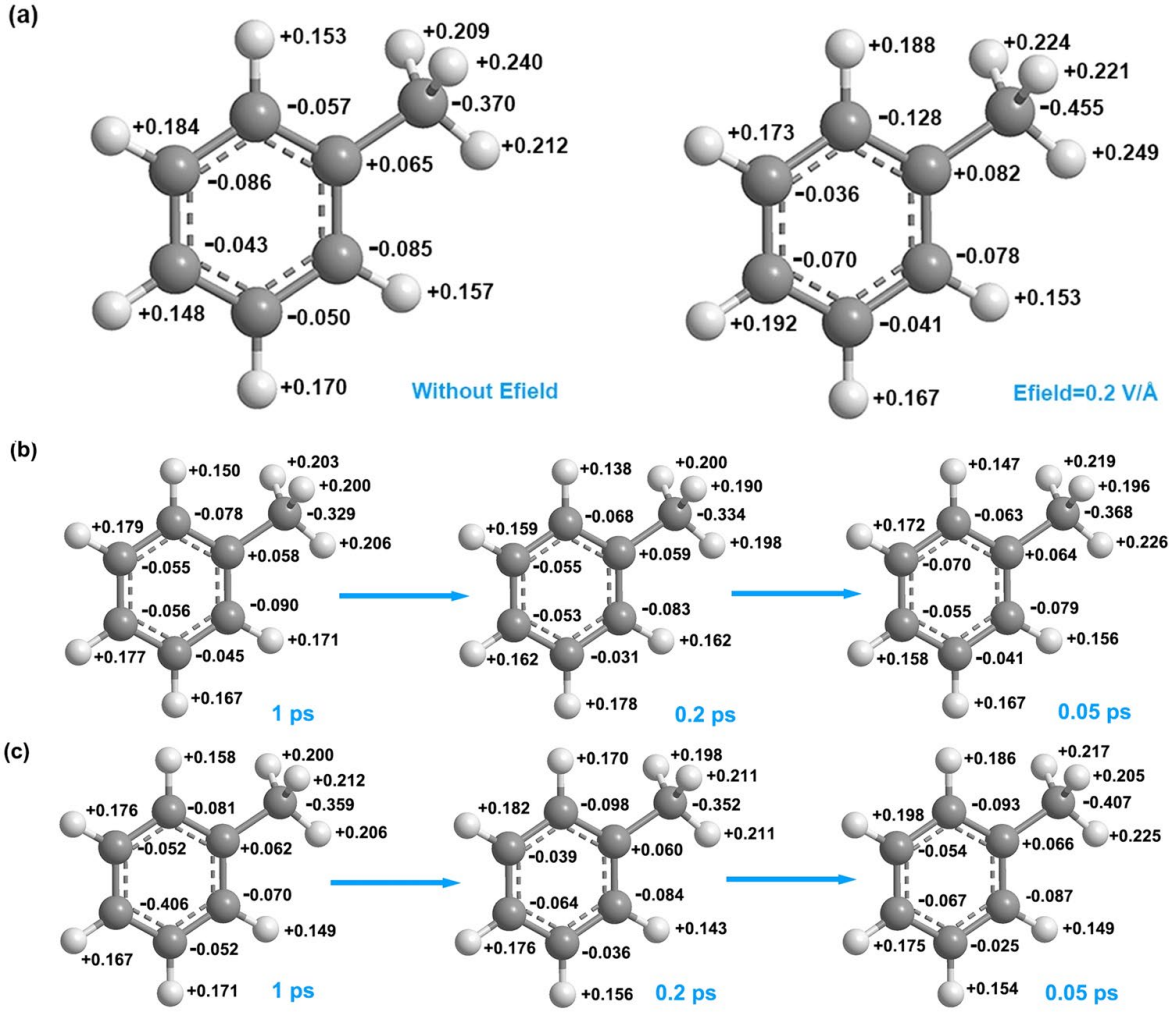
Partial charge of atoms in toluene molecules during ReaxFF NVT-MD simulations. (**a**) simulations with/without Efields at the transition state. (**b**) 1 ps time evolution of partial charge in simulations without Efields before transition state. (**c**) 1 ps time evolution of partial charge in simulations with Efield = 0.2 V/Å before transition state.

Field-induced polarization^38, 39^ often happens when a molecule is in a Efield. This polarization may be critical for certain chemical reactions. To study the field-induced polarization of toluene in Efields, we calculated the average partial charges of atoms in toluene at the transition states for simulations with/without Efields. The toluene configuration right before its decomposition was defined as its transition state. The partial charges of atoms in toluene at the transition states are shown in Fig. 11(a), which represents the situation for four out of ten simulation runs. This layout of partial charges suggests that Efield may enhance polarization of toluene before its decomposition. However, the other six simulation runs show layouts of partial charges was not relevant to the existence of Efields. Similarly, when we examined the partial charges of atoms in toluene and right before the transition states, in some cases, the enhanced polarization of toluene can be observed, for instance, as shown in Fig. 11(b) and (c). However, there are other situations where the effect of Efields is not obvious at all. (Results are no shown here) This observation could be due to the charge equilibration (QEq)^40, 41^ method used in the simulations. While the method is very useful for simulations without the existence of Efields, it is still not sufficient for current studies where Efields plays a key role in chemical reactions. With the further development of this method, such as the polarizable charge equilibration method (PQEq), the polarization of toluene during its oxidation will be better described to reveal more details of the effects of Efields.

## Discussion

By taking the oxidation of toluene as a model system, the possible effects of Efields on chemical reactions were examined at various Efield strength, reaction temperature, and toluene density with ReaxFF MD simulations. Results show that the initial reaction time of toluene is reduced remarkably in Efields. Stronger strength of Efields leads to a faster oxidation rate of toluene. Further studies reveal that Efields may initiate the oxidation of toluene at 2100 K which is otherwise not possible to happen when Efields are not at present. With Efields, the oxidation rate of toluene at 2100 K is comparable with the oxidation rate of toluene at 2900 K when Efields are not applied.

Two representative reaction pathways of toluene were observed during its oxidations, one starts with a hydrogen abstraction reaction, the other starts with a pyrolysis reaction. Both pathways were previously reported in other experimental and kinetic modeling studies. The Efields employed in this study significantly enhance the occurrence of the initial radical generation for these two pathways and lead to a shorter initial reaction time and a faster decomposition of toluene, However, it does not seem to favor any of the pathways.

Finally, Efields do not seems to have enhanced the polarization of toluene during its transition state. The partial charges of atoms in toluene is largely determined by the relative positions of the atoms. This suggests that a polarizable QEq method is needed to take the effects of Efields into account.

## Methods

The ReaxFF for C/H/O system developed by van Duin *et al*.^17^ was employed to determine the effects of Efields on the oxidation of toluene. A series of 10 periodic systems each containing 1 toluene and 100 oxygen molecules was created in cubic boxes. Each system contains one toluene molecule. The use of a single toluene molecule not only allows the detailed evaluation of the chemical events associated with the oxidation process but also avoid the complication due to the competitive reactions between intermediates when multiple toluene molecules are involved^25^. The densities of the systems were set by the edge lengths of the boxes. Each system has a different initial configuration. An example of the initial configurations was shown in Fig. 1a.

The ReaxFF MD simulations were performed with a constant number of atoms (N) in a constant volume (V) and temperature (T), referred to as NVT-MD. Each system was first energy minimized and then equilibrated for 50 ps using NVT-MD simulation at 5 K with a time step of 0.1 fs. This time step is recommended for hydrogen oxidation at high temperature to investigate the chemical reaction^18^. It gives reasonable descriptions for the oxidation reaction of hydrocarbons. During the equilibration simulations, the C-O and H-O bond parameters were switched off to prevent reactions from occurring during the equilibration of the system. All these equilibrated systems were then used in NVT-MD simulations for 3000 ps run using a Berendsen thermostat^42^ where the bond-order cutoff is 0.3, the MD time step is 0.1 fs, and the damping constant is 0.1 ps to avoid the obvious temperature fluctuation. Table 1 lists details of the Efield applied in the Z direction to the systems. Ten initial configurations were examined for each individual Efield to obtain statistically meaningful information. To investigate the effect of temperature on toluene oxidation, a series of NVT–MD simulations were performed at 2100, 2300, 2500, 2700, 2900, and 3100 K, respectively. In addition, another three scenarios were examined. Each scenario have a different combination of temperature and Efield, as shown in Table 1. This allows us to compare the effect of temperature and Efield at various conditions. For each simulation, the dynamic trajectories of toluene oxidation, which include atomic positions and velocities, were saved for every 0.1 ps. The bond-order cut off values for each pair of elements used for molecular recognition about the intermediates and products formed during the ReaxFF simulations can be checked elsewhere^25, 43, 44^. The LAMMPS software package was used to perform all the simulations.

**Table 1:** Details of ReaxFF MD simulations.

Efields (V/Å)	Toluene/Oxygen	Temperature (K)	Density (g/cm^3^)	Ensemble
2 × 10^−5^–2 × 10^−1^	1/100	2100–3100	0.15–0.35	NVT
0 (control)	1/100	2100–3100	0.15–0.35	NVT
